# Role of Nanodiamonds in Drug Delivery and Stem Cell Therapy

**DOI:** 10.15171/ijb.1320

**Published:** 2016-09

**Authors:** Shakeel Ahmed Ansari, Rukhsana Satar, Mohammad Alam Jafri, Mahmood Rasool, Waseem Ahmad, Syed Kashif Zaidi

**Affiliations:** ^1^Center of Excellence in Genomic Medicine Research, King Abdulaziz University, Jeddah-21589, Kingdom of Saudi Arabia; ^2^Department of Biochemistry, Ibn Sina National College for Medical Sciences, Jeddah-21418, Kingdom of Saudi Arabia

**Keywords:** Biomedical applications, Drug delivery, Hydrogels, Nanodiamonds, Surface functionalization

## Abstract

**Context:**

The use of nanotechnology in medicine and more specifically drug delivery is set to spread rapidly. Currently many substances are under investigation for drug delivery and more specifically for cancer therapy.

**Evidence Acquisition:**

Nanodiamonds (NDs) have contributed significantly in the development of highly efficient and successful drug delivery systems, and in stem cell therapy. Drug delivery through NDs is an intricate and complex process that deserves special attention to unravel underlying molecular mechanisms in order to overcome certain bottlenecks associated with it. It has already been established that NDs based drug delivery systems have excellent biocompatibility, nontoxicity, photostability and facile surface functionalization properties.

**Results:**

There is mounting evidence that suggests that such conjugated delivery systems well retain the properties of nanoparticles like small size, large surface area to volume ratio that provide greater biocatalytic activity to the attached drug in terms of selectivity, loading and stability.

**Conclusions:**

NDs based drug delivery systems may form the basis for the development of effective novel drug delivery vehicles with salient features that may facilitate their utility in fluorescence imaging, target specificity and sustainedrelease.

## 1. Context

Nanoparticles mediated drug delivery has attracted the attention of researchers for safely transferring the drug of choice to the site of interest in a biological system apart from ensuring their biocompatibility (1,2). These nanoparticles prevent high systemic loads of drugs at specific targeted sites that usually results in manifestation of unwanted toxicity. The process of nanoparticle based drug delivery occurs either by delivering drugs into the cytoplasm or through extracellular domains of transmembrane signaling molecules (3). Since human digestive tract contains greater acid/enzyme content, it degrades protein and peptide drugs before they are absorbed into the bloodstream. Therefore, acid labile drugs need adequate protection against unfavorable acidic environment of the gut. Apart from this, several charged compounds and macromolecules possess weak partitioning properties which make them poorly absorbed through biological membranes. Hence the need of nanoparticle based drug delivery systems arises which can inhibit acid and peptidase mediated degradation thereby allowing sufficient time for the drug to be absorbed via gastrointestinal tract epithelium (4). Additionally, nanoparticle based delivery vehicles may be exploited to release pharmacologically relevant drug concentration at specific target sites such as malignant tumors.

## 2. Evidence Acquisition

### 2.1. Benefits and Limitations of Existing Nanoparticle Based Drug Delivery Platforms

During past couple of decades, several studies have significantly contributed for the development of novel nanoparticle based platforms with different compositions and biological properties for plethora of drug and gene delivery applications (5-7). Quantum dots, chitosan and polylactic/glycolic acid based nanoparticles have also been suggested for *in vitro* RNAi delivery (8). The brain cancer is one of the most difficult to manage malignancy mainly because of the inability of therapeutic agents to pass through the blood brain barrier (BBB) and reach brain tumor, therefore, loperamide and doxorubicin were successfully bound to nanomaterials cross the intact BBB in order to release them in desired therapeutic concentrations to the brain (9). Moreover, peptide-based nanotubes were also exploited to target vascular endothelial growth factor receptor and cell adhesion molecules like integrins, cadherins and selectins, as a new therapeutic approach to control disease progression (10).

Inspite of observed promising benefits of nanoparticle based drug delivery platforms in addressing numerous key issues associated with current clinical practice for the treatment of difficult to treat diseases, there are certain well-known challenges in translating this exciting technology for the clinical application. The potential toxicity of nanoparticles remains one of the major concerns in the field of nanoparticle based drug delivery. Therefore, an in-depth knowledge about the nature and mechanism of nanoparticle-induced toxicity is required to facilitate their successful clinical translation. For example, their toxicity on central nervoussystem and their interactions with the cells and tissues are poorly understood (11). Furthermore, nanoparticles usually have short circulation half-life due to faster elimination through opsonization by phagocytic cells inside the human body. Hence, the nanoparticle surfaces need to be modified in a way to avoid phagocytosis by circulating macrophages.

Recent years have witnessed significant increase in the application of carbon based nanoparticles in the field of biomedicine and electronics (12) which has led to the emergence of NDs as novel constituents of carbon based nanoparticles. They have excellent biocompatibility, photostability and facile surface functionalization properties which could be utilized to promote longer circulation half-lives, to improve drug pharmacokinetics and reduce side effects of therapeutically active substances. These outstanding features of NDs might also help in devising drug-delivery vehicles with the ability to overcome the action of drug efflux pumps, thus minimizing cancer chemo- resistance and maximizing the therapeutic efficacy of the anticancer drugs (13-14).

### 2.2. Nanodiamonds (NDs)

NDs are carbon-based nanomaterials that provide large surface area. They can be functionalized with different ligand molecules, which can be used to conjugate various compounds or drugs (15). Same as bulk diamond, the basic crystal structure of an ND consists of a nanocrystal having tetrahedral bonded carbon atoms in the form of a three-dimensional cubic lattice which imparts the properties of diamond and an onion shaped carbon shell containing a coat of functional groups on the surface (16,17). However, ND is often described as a crystalline diamond core surrounded by an onion like amorphous graphite shell (18). The sp^2^/sp^3^ bonds in ND are quite flexible endowing it with the ability to assume two geometrical forms for example, the stretched face of diamond can behave as a graphene plane whereas the puckered graphene may become a diamond surface. This unique characteristic feature of ND particles promises a greater degree of template flexibility particularly around the curved surface where electrons are unstable (19,20).

NDs can be easily doped with nitrogen and possess nitrogen-vacant (NV) defect centers in their crystal lattice which make them suitable as photoluminiscent probes for several *in vivo* and *in vitro* applications (15). The presence of the NV centers - a nitrogen atom next to a vacancy in NDs, leads to useful fluorescence properties. They emit bright fluorescence at 550-800 nm from NV centers produced by high-energy ion beam irradiation and subsequent thermal annealing. The excellent emission property, together with negligible cytotoxicity and easiness of surface functionalization, makes NDs a promising fluorescent probe for single-particle tracking in heterogeneous environments as well as using them as biomarkers and in biolabelling studies (21). Research activities concerning the bioimaging and drug delivery applications of NDs have also been discussed in detail by previous researchers (22). The focus was on the prospective of using NDs in bioimaging, their interaction with biological objects and possibility of modifying their surface by attaching biological/medical molecules for drug delivery applications (23).

**Figure 1:**
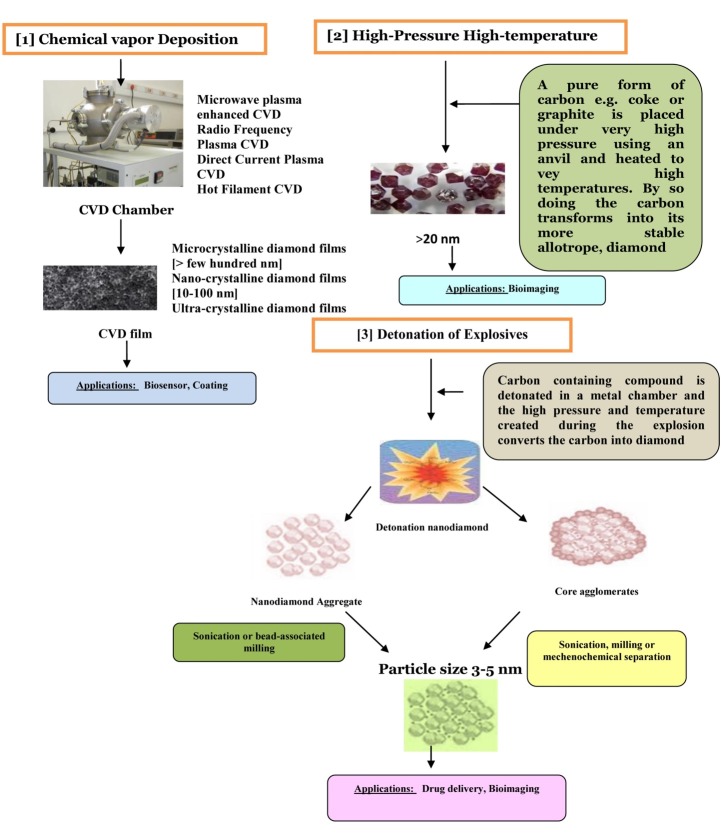
Classification of nanodiamonds on the basis of synthesis method (25). CVD is a technique used to produce thin films of solid materials. The method involves high temperature heating of substrates and then exposing them to precursor materials in gaseous state. The precursors are deposited on the substrate surface to yield a coating of required material. High-Temperature-High-Pressure method is employed for highly controlled synthesis of nanoparticles. The detonation method is suitable for bulk synthesis of diamond nanoparticles. This method uses high-pressure-high-temperature conditions produced by shock wave created during detonation of explosives

Several studies suggested that NDs can be easily functionalized by covalent and non covalent method, and their biocompatibility is superior over other carbon containing nanomaterials like single walled and multi walled nanotubes, and carbon blacks (24). They have been classified on the basis of their particle size and on the method of synthesis (Figure 1).

### 2.3. Surface Functionalization of NDs

**Figure 2:**
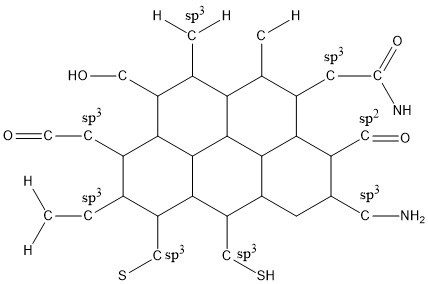
Schematic representation of Nanodiamond crystal structure showing several functional moieties

The surface of NDs can be modified by many functional groups for imparting extra stability (Figure 2).

These surface modifications can be achieved through covalent and/or non-covalent bonds with different methods, though each method has its own advantags and disadvantages. Covalent modifications of NDs have resulted in stable drug complexes. However, the used processes are typically complex (26,27), suffering from difficulty in toxic solvents removal introduced during preparation, and inconsistent slow release of the drug. On the contrary, drugs can be attached to NDs easily by non-covalent methods, however with lower stability. Loading of drug by this method can be modulated simply by controlling concentration of involved inorganic molecules such as NaOH and NaCl. This method also imparts slowrelease of drug into the cells, thereby reducing the possibility of undesired side effects of drug. Therefore, at present, non-covalent method is preferably chosen to build ND-based drug delivery systems. Arnault has also discussed the control of the surface chemistry of NDs for improving their colloidal behavior (28). NDs possess high surface free energy, which allows to form clusters of about 10-100 nm even when they are dispersed in a solution. Additionally, numerous functional molecules and variety of drugs have been attached successfully on their surface by non-covalent interactions (29). NDs can be embedded in a silica shell terminated by a hydrophilic, azide- or alkyne-substituted PEG polymer. It was observed that in contrast to unmodified NDs, this nanoarchitecture brings high colloidal stability in buffers and biological media. Furthermore, such NDs allow the possibility of modifying particles by biomolecules in high yields using click chemistry (30,31).

Hydrophilic moieties containing chlorine, ketonic and carboxylic group have been introduced earlier on the surface of NDs by a thermal induction and plasma treatment method (32-34). Moreover, covalent attachment was also obtained onto NDs by using alkyl chains, fluorine and silicon (35-37). Surface modified ND films were used to adsorb small molecules like allyl alcohol, sulfonic acids, thiols, and complex structures like DNA and enzymes in preparing sensors and for the delivery of therapeutic agents (38-42). The predominant functional moieties present on the surface of NDs include carboxylic (COOH) and ketonic (C=O) groups apart from having alcoholic and ether groups (43-46). Moreover, amine group was introduced on the surface of NDs by reacting chlorinated diamond with gaseous ammonia at elevated temperature, using submicronparticles by several investigators (47,48). In another novel approach, ultra-bright fluorescent NDs were developed and their thermal and kinetic optimum of nitrogen-vacancy (NV) center formation was identified (49). The presence of NV centers imparted excellent biocompatibility, bright fluorescence and high magnetic sensitivity at ambient conditions. These fluorescent NDs have great potential in designing nanosensors (50). The core/shell structures of NDs surrounded by porous silica shells have demonstrated a remarkable increase in drug loading efficiency. Additionally, such NDs decorated with carbon dots have shown excellent potential as bioimaging probes (51). Attachment of various drugs to NDs has been listed in Table 1.

**Table 1:** Ligands used to modify the surface of NDs

**Ligands**	**Applications**	**References**
Carboxylic acid	Adsorption of mycotoxins, aflatoxin B1 and ochratoxin A	(57,58)
Benzoyl peroxide and dicarboxylic acid	Binding anticancer drugs	(59)
Fluorine	Development of ND based bioconjugates for labeling, drug delivery and other applications	(60-63)
Lysine	Development of non-viral vectors for transferring genetic materials across cellular membranes	(64,65)
Alkyl lithium, ethylenediamine, glycine ethyl ester generated alkyl	Biolabelling and specific binding of drugs for efficient drug delivery Enzyme immobilization	(47)
Glutaraldehyde	Chemical applications	(66)
Thiolated	Therapeutic and drug delivery applications	(67)
CO, OH, and NH-groups	Binding protein drug with excellent drug release properties	(68-70)
N,O-carboxymethyl chitosan	Production of highly dispersed suspensions of detonated NDs in polar media directly from dry powders	(71)
Hydrocarbon and Perfluorobutyl radical	Chemical applications	(72,73)
Hydrogenation and hydroxylation	Analytical applications	(58)
Hydrogen	Integration of epoxy polymer composites for corrosion resistant coatings, as well as biomedical applications by attaching complex molecules such as	(74,59)
Chlorine, Glycine	proteins and DNA	(47)

Alarge number of covalent and non covalent methods have been used in past to modify NDs. Surface modification of NDs by N,O-carboxymethyl chitosan and benzoquinone to assist the binding of drug on these modified NDs via non-covalent methods have been reported elsewhere. The results were illustrative of lower cytotoxicity and absence of photo-bleaching. Furthermore, performing *in vivo* studies, biological imaging, drug delivery and other theranostic applications have been envisaged (52-55). Contrarily, Liu *et al*. (56) reported the attachment of paclitaxel to surface modified NDs prepared by covalent method. It was observed that application of 0.1-50 μg.mL^-1^ modified ND based paclitaxel significantly decreases the cell viability of A549 human lung carcinoma cells by inducing mitotic arrest and apoptosis, consequently blocking the tumor growth.

## 3. Results

### 3.1. In vitro and In vivo Toxicity Studies of NDs

Negligible cytotoxicity has been reported for NDs with little changes in gene expression under physiological conditions (75). No significant changes were observed in the expression of TNFαand Bcl-x genes after incubation with the acid purified NDs when compared with controls. Their therapeutic potential was confirmed by observing ND-induced morphological changes in HT-29 human colorectal adenocarcinoma cells and effect on cell viability on murine macrophages (75). Chow and co-workers noticed that adsorption of doxorubicin was increased significantly on NDs after addition of NaCl (10 mg.mL^-1^) to the reaction system. Moreover, NDs bound doxorubicin exhibited less toxicity on RAW264.7 mouse macrophage cell line and HT-29 human colon cancer cells as compared to doxorubicin alone. The possible explanation might be that NDs provided a protective effect to doxorubicin by favoring the slow release of drug and preventing the normal cells from its side effects. They also used this drug complex for the treatment of liver and breast cancer, and observed significant killing of drug resistant cells at relatively lower dosages, resulting in the decreased toxicity of the drug . Doxorubicin was released from this complex steadily and exhibited increased retention in blood circulation and tumor causing enhanced inhibition of tumor growth as compared to usual doxorubicin treatment in tumor models (76). These studies provide a promising foundation to find ND-based drug development and their potential clinical applications.

It was shown that optimum binding of doxorubicin on NDs occurs only at high pH and availability of at least 10% of the ND surface area (77). A comparative study reported that NDs were significantly non-toxic to macrophages and neuroblastoma cells, preventing the formation of reactive oxygen species as compared to carbon black, multi-walled carbon nanotubes and single-walled carbon nanotubes (78,79). In another study, A549 lung cancer cells and 3T3-L1 embryonic fibroblasts were incubated with NDs for 10 days, and no adverse effect on cell or gene expressions was observed in cancer cell lines during their progression and adipogenic differentiation (68). Vaijayanthimala *et al*. (12) observed that NDs can be readily taken up by 3T3-L1 pre-adipocytes, HeLa cancer cells and 489-2 osteoprogenitors cells in large amount suggesting their greater biocompatibility. Ease in the adsorption of doxorubicin (anticancer drug), purvalanol A (drug for liver cancer), hydroxytamoxifen (drug for breast cancer), dexamethasone (anti-inflammatory drug), insulin (drug for diabetes) and TAT (a cell penetrating peptide) onto NDs have been reported, preventing premature release and improving therapeutic efficacy (76,80-83).

The toxicity of fluorescent NDs (FND) in long term *in vivo* imaging was evaluated in *C. elegans* (84). FNDs were given to *C.elegans* either by feeding with colloidal FND solution or microinjecting FND suspension into the gonads. The toxic potential of FND was ascertained by assessing its effect on longevity and reproductive potential. The result obtained in study emonstrated that FNDs were devoid of any toxicity and did not cause evident stress to the worms. FNDs were found to be highly efficient in continuous imaging of entire alimentary canal and mapping of several biological processes including development of the living organism from fertilized egg for several days.

### 3.2. Application of NDs in Drug Delivery System

A new drug delivery system consisting of NDembedded in contact lens (for drug sequestration and sustained release) was tested using timolol maleate (drug used for treating ocular disease, glaucoma) in the presence of lysozyme in trabecular meshwork cells (85). This study established that the ND-embedded lenses can be utilized for drug sequestration, controlled release and enzyme activation purposes. In another study, NDs were used to deliver an anti-cancer drug 10-hydroxycamptothecin (HCPT) in the presence of NaOH (pH 8.2) to enhance the adsorption of HCPT on NDs. It was determined that NDs-HCPT complex showed slow and steady release of the drug under slightly acidic condition and was highly effective on tumour cells as compared to free HCPT (86). Similar studies were also carried out by adsorbing cisplatin onto NDs. Contrarily, the drug in this case was released at high rate in the presence of PBS buffer (pH 6) from the ND complex (87). It should be noted that subcutaneously injected NDs for three months in a mouse model did not elicit any inflammatory response (88). Xing *et al*. (89) observed improved expression of DNA repair proteins, p53 and MOGG-1 when embryonic stem cells were challenged with NDs. Bakowicz and Mitura (90) also reported the absence of adverse immune response in rats following 10 days of intraperitoneal injection of NDs. These studies suggested excellent biocompatibility of NDs as they did not evoke any inflammatory immune response at injection site when administered through various routes in animal models.

Several molecules like insulin, cytochrome c, lysozyme, apoobelin were immobilized on NDs by non-covalent method to improve their functionality (91-94). In another study, *L*-polylysine and polyarginine were used to produce their respective ND conjugates containing greater number of primary amino groups (12,95). Polyaniline, polyethyleneimine and poly (lactic acid) were also used to functionalize NDs by amide bond formation (96-99). Moreover, Sulfur, thiols and sulfonic acids were also used for modifying NDs in preparing plasmonic structures and for conjugating very complex (bio) molecules efficiently (100-102). An excellent review has appeared recently dealing with all the possible transformations of NDs in great detail (103).

Carbon nanoparticles possess small size, penetratethrough the cells easily and can be surface functionalized. These properties enabled their application in designing drug delivery vehicles for attaching small molecule drugs and peptides (104-107). However, carbon nanotubes and graphene are the two forms of carbon based nanoparticles that showed cytotoxicity, oxidative stress and apoptosis (108-110). Thus, the need arises to have safer drug delivery systems that employ surface modified nanoparticles. The NDs are such versatile tools for building drug delivery systems owing to their chemical inertness, optical transparency, robust hardness high specific area, and excellent biocompatibility. Promising data, apart from low toxicity and high cellular uptake, have been reported for use of NDs in complete cell culture media (111). NDs, as a drug carrier, can be used as a thin film for efficient interaction of drug, and in the form of spontaneous clusters known as ND hydrogel that favors attachment of drug. It should be noted that ND based films can be introduced immediately after surgical removal of a tumor to target residual cancerous cells to prevent the tumor from recurring. Thus, they have gained extensive attraction in developing highly efficient drug delivery systems for treating superficial tumors such as skin, breast, head and neck cancers, and superficial skin inflammations where the drug is delivered transdermally to tumors or inflammation sites thereby reducing the toxicity on normal tissues. Table 2 shows the list of studies where NDs were used to develop drug delivery systems.

**Table 2:** Ligands used to modify the surface of NDs

**Drugs**	**Applications**	**References**
Doxorubicin	Treatment of colorectal carcinoma	(75,76,80,86,24,121)
10-hydroxycamptothecin (HCPT)	Intracellular transportation of chemotherapeutic drug	(86)
Paclitaxel	Drug delivery and cancer therapy for lung carcinoma	(56)
Insulin	For delivering insulin and other therapeutic treatments	(81)
Purvalanol A	Treatment of liver carcinoma	(80)
4-hydroxytamoxifen	Treatment of breast carcinoma	(80)
Si RNA	*in vivo* imaging	(84)
polymyxin B	Development of drug delivery platforms	(24)
Epirubicin	Overcoming chemoresistance in hepatic cancer stem cells	(128)

The simplest way to develop ND films was achieved by self-assembly process in which ND hydrogels were fixed on poly-lysine coated glass substrate and the thickness of ND-poly-lysine film was obtainedthrough layer-by-layer technique (112-115). The films thus formed could assemble several biological molecules. The cellular gene expression studies and MTT assays revealed excellent biocompatibility and safety of the ND films. The self-assembled doxorubicin on ND films showed anti-inflammatory effects as well as slow and controlled release when tested against RAW264.7 cells apart from demonstrating significant reduction in toxic side effects on the normal tissues. ND bound doxorubicin sandwiched in a Parylene C polymer microfilm was found to be stable; slowly released even after a month due to the potent sequestration ability of the doxorubicin-ND complex and the release modulating nature of the thin Parylene layer (116). Moreover, Xi *et al*. (117) utilized NDs to enhance delivery of DOX in a preclinical glioma model using a convection-enhanced delivery method and demonstrated remarkably enhanced efficacy of doxorubicin. It was found that NDs markedly enhanced DOX uptake and retention in glioma cells. Thus, ND-DOX delivered by this method was significantly more efficient in killing tumor cells than uncomplexed DOX.

### 3.3. Applications of NDs Based Hydrogel

The research on NDs based hydrogels has mainly concentrated on their evaluation as a vehicle for targeted transportation, controlled release, declined drug concentrations and reduced side effects. This material also provides better penetration of the drug complex to gain access to the interior of the cells. Some of the improvements using NDs based hydrogels for drug delivery are as follows. Zhang *et al.* (107) reported heterofunctionalization of a 2-8 nm ND for a drug delivery system. This multifunctional system has simultaneous capabilities of targeting, imaging and enhancing therapy. ND based hydrogels were conjugated with the anticancer cell proliferation-inhibiting and apoptosis-stimulating paclitaxel-DNA via fluorescently labeled oligonucleotide strands, and with anti-EGFR monoclonal antibodies. The feasibility of these conjugates was studied on human breast cancer cell lines (MDAMB-231 and MCF7). Fluorescently labeled oligonucleotide linkers enabled the intracellular observation and quantification of resultant ND conjugates and anti-EGFR monoclonal antibodies provided the internalization and delivery of anticancer agents into EGFRoverexpressing cells. The results showed enhanced cellular internalization and therapeutic activity of covalently attached chemotherapeutic and targeting moieties on ND surface. Liu *et al.* (1118) also successfully covalently linked 3-5 nm sized NDs with paclitaxel for drug delivery and cancer therapy. The results showed that ND-paclitaxel significantly reduced the cell viability and induced mitotic arrest, apoptosis and anti-tumorigenes in A549 (human lung carcinoma) cells, thereby inducing cell death in A549 cells. Furthermore, ND-paclitaxel markedly blocked the tumor growth and formation of lung cancer cells in xenograft SCID mice.

### 3.4. NDs in Stem Cells

Stem cells have increasingly become the focus of attention in translational research as well as in the development of human cellular therapy for difficult to treat diseases. There is an urgent need for the development of novel and improved methods to tract the fate of a specific stem cell under *in vivo* conditions. A significant amount of data is available to support the fact that nanoparticles including NDs can be utilized for tracking stem cells *in vivo* without affecting their normal biology. It has been demonstrated that the presence of fluorescently labeled superparamagnetic iron oxide particles (~0.9 μm) and carboxylated NDs (~0.25 μm) did not adversely affect normal morphology, osteogenic and adipogenic differentiation potential, CD marker expression, cytokine secretion and other biochemical parameters of adipose-derived mesenchymal stem cells (119). In addition, several studies have clearly established excellent biocompatibility of NDs in a variety of cell lines without noticeable cytotoxicity (88). However, it has also been reported that NDs slightly stimulate expression of DNA repair proteins such as p53 and MOGG-1 in embryonic stem cells suggesting DNA damage. Furthermore, it was observed that oxidized NDs have higher potential to cause DNA damage compared to raw NDs, possibly due to specific surface chemistry. The lung stem cells are being extensively considered for the treatment of lung diseases particularly as regenerative therapy for repairing damaged or lost lung tissues in patients. Therefore, accurate tracking of stem cells under *in vivo* conditions constitutes an important step in the process of understanding how stem cells differentiate and regenerate themselves. Numerous optical methods and photo-probes have been employed for this purpose but most of these methods are limited due to undesired photo-bleaching, toxicity and interference from background tissue. Wu *et al*. (120) have demonstrated that FNDs can be utilized to track and identify engrafted lung stem/progenitor cells by recognizing CD45–CD54^+^CD157^+^ surface markers employing fluorescence-activated cell sorting or fluorescence lifetime imaging microscopy techniques in order to evaluate their regenerative capabilities. It was also observed that FND labelling did not interfere with normal stem cell properties including self-renewal and differentiation into functional pneumocytes. The NDs have also been used for target-based delivery of drugs in the stem cells. For instance, epirubicin, an anthracycline anticancer drug attached to NDs was delivered in hepatic cancer stem cells to overcome chemoresistance. It was observed that epirubicin bound to NDs created a rapidly synthesized and stable NDs-epirubicin complex that promoted the endocytic uptake and enhanced tumor cell retention. These attributes mediated effective killing of both cancer stem cells and non-cancer stem cells *in vitro* and *in vivo* (121).

## 4. Conclusions

Owing to excellent biocompatibility, bright photoluminescence based on color centers and outstanding photostability, NDs could provide much needed foundation to develop application-specific drug delivery vehicles to treat various cancers. This can be achieved by preparing NDs in a systematic manner by combining disciplines such as nanomanufacturing, drug discovery and molecular biology/medicine which will serve as collaborative roles in downstream realization of clinical ND. Moreover, the studies showed that the surface of NDs can be modified by several functional groups via covalent and non-covalent methods for imparting extra stability to them. Hence, in near future, NDs based drug delivery systems can provide hope for effective treatment of aggressive diseases and treating resistant diseases in large. The progress in the development of a number of methods of ND functionalization with different kinds of drugs will definitely open possibilities to apply constructed systems not only of drugs but also gene and protein delivery. Moreover, continuous research on NDs is needed to seek further development of including magnetism (FRET and magnetic resonance) and nonlinear optical properties (optical trapping) in NDs so that they can be successfully employed for bioimaging, labeling and drug delivery systems.

## 5. Future Perspective

There has been a considerable progress in the applications of ND in the field of biomedical sciences including bio-imaging and targeted drug delivery in the past few years. However, some bottlenecks still exist that have to be overcome. For medical (clinical) applications, size of NDs is a limiting factor. Aggregation of NDs is a serious problem, especially for sizes smaller than 50 nm. Therefore, an effective method is needed to prevent cluster aggregation. Majority of the current studies have mainly used cellular models or microorganisms for their experiments. The interaction of NDs with animal organs and the fate of ND/drug clearance in the animal body have not been systematically studied. If ND-based drug delivery is to be converted into successful clinical application, well designed comparative studies using important drug delivery methods are also warranted. Research on NDs continues to seek further development of novel methods such as addition of magnetism and nonlinear optical properties such as optical trapping, FRET and magnetic resonance in NDs. These properties will aid in successful validation of bioimaging, labeling and drug delivery tracing using NDs for future biomedical applications.

## Acknowledgements

The authors are thankful to Dr. Peter Natesan Pushparajan for making the first figure of the manuscript.

## Authors’ Contributions

Study concept and design: Shakeel Ahmed Ansari and Rukhsana Satar. Acquisition of data: Rukhsana Satar and Mohammad Alam Jafri. Drafting of the manuscript: Rukhsana Satar, Syed Kashif Zaidi and Mahmood Rasool. Critical revision of the manuscript for important intellectual content: Waseem Ahmad and Mohammad Alam Jafri.
